# Disease burden of cervical cancer in China from 1990 to 2023 and prediction of future trends

**DOI:** 10.3389/fonc.2026.1802330

**Published:** 2026-05-13

**Authors:** Tingzhou Yan, Qihao Ma, Haoxin Shi, Ding Qi, Li Liu

**Affiliations:** 1 Heilongjiang University of Chinese Medicine, Harbin, China; 2 The First Affiliated Hospital of Heilongjiang University of Chinese Medicine, Harbin, China; 3 Second Affiliated Hospital, Heilongjiang University of Chinese Medicine, Harbin, China

**Keywords:** ARIMA model prediction, cervical cancer, disease burden, GBD database, joinpoint regression analysis

## Abstract

**Objective:**

To analyze the temporal trend of the disease burden of cervical cancer in China from 1990 to 2023 and to forecast incidence and mortality trends from 2024 to 2038, thereby providing data support and a scientific foundation for optimizing cervical cancer prevention and control strategies in China.

**Methods:**

Data from the Global Burden of Disease (GBD) 2023 database were used to extract incidence, prevalence, mortality, and disability-adjusted life years (DALYs) related to cervical cancer in China. Age-standardized rates (ASRs) were applied for comparative analysis. Temporal trends were evaluated using Joinpoint regression, and an autoregressive integrated moving average (ARIMA) model was employed to project the age-standardized incidence rate (ASIR) and age-standardized death rate (ASDR) from 2024 to 2038.

**Results:**

Between 1990 and 2023, absolute numbers of prevalent cases, deaths, and new cases increased, whereas all age-standardized rates declined. ASDR decreased by 52.5%, and the age-standardized DALYs rate decreased by 54.9%. The disease burden was concentrated in women aged ≥55 years. Joinpoint regression showed an overall decline in ASIR (AAPC = –1.02%), with rebounds during 2013–2017 and 2020–2023. ARIMA projections indicate that ASIR will gradually decline from 11.14 per 100,000 in 2024 to 8.70 per 100,000 in 2038, a cumulative reduction of 21.9%, while ASDR will drop sharply from 3.70 per 100,000 to 0.48 per 100,000, a cumulative reduction of 87.0%.

**Conclusion:**

The diverging trends between declining age-standardized rates and rising absolute numbers indicate a critical transition in China’s cervical cancer control efforts. While mortality reduction has been substantial, the growing patient population—particularly among older women—demands a strategic shift toward integrated prevention. Future efforts should expand HPV vaccination coverage, optimize screening for under-screened populations, and strengthen health system capacity to manage the increasing absolute burden. These findings provide an evidence−based foundation for advancing China’s cervical cancer elimination strategy.

## 1 Introduction

Cervical cancer ranks as the fourth most common malignancy regarding incidence and mortality among women worldwide, China bears a disproportionate share, accounting for 18% of global cervical cancer incidence and 17% of global mortality (1). Due to its large population, cervical cancer imposes substantial economic burdens on Chinese society. In many developing and underdeveloped regions of China, economic conditions, cultural factors, and uneven healthcare resource distribution vary widely. These variations contribute to persistently high cervical cancer incidence and mortality rates (2). The etiology of cervical cancer is closely linked to infection with human papillomavirus (HPV), a circular double-stranded DNA virus infecting skin and mucosal epithelia, with 222 subtypes currently identified (3). Among these, 12 subtypes are classified as high-risk HPV (HR-HPV) by the International Agency for Research on Cancer. Persistent HR-HPV infection significantly increases the risk of cervical carcinogenesis, leading to cervical squamous intraepithelial lesions (SIL) that can progress through breaking the subepithelial basement membrane, invading underlying stroma, culminating in cervical cancer (4). At present, comprehensive analyses of the disease burden and future trends of cervical cancer among Chinese women remain limited. To support the establishment of a robust cervical cancer prevention and treatment system and accelerate cervical cancer elimination, this study utilizes the GBD database to perform a comparative analysis of cervical cancer’s disease burden in China from 1990 to 2023 and to project its epidemiological trends from 2024 to 2038, thereby informing evidence-based prevention strategies to reduce the disease burden.

## 2 Data and methods

### 2.1 Data source

The data utilized in this study were obtained from the GBD 2023 dataset released by IHME at the University of Washington, USA. This database presents a comprehensive assessment of risk factors for 375 diseases and injuries across 204 countries and territories from 1990 to 2023 (5). The IHME integrates numerous high-quality data sources—including national death registries, hospital records, and disease surveillance systems—and applies standardized modeling methods to estimate disease indicators by region, age, and sex, ensuring consistency and comparability. Chinese data primarily derive from the Disease Surveillance Points and Vital Registration systems maintained by the Chinese Center for Disease Control and Prevention, thus guaranteeing data reliability. This dataset is publicly accessible via the Global Health Data Exchange platform (http://ghdx.healthdata.org). Cervical cancer cases were defined using the International Classification of Diseases, 10th Revision (ICD-10) code C53 (malignant neoplasm of cervix uteri), corresponding to ICD-9 code 180. To eliminate the confounding effect of age structure differences across populations and over time, all rates were age−standardized using the GBD world population standard, as implemented by IHME. This standard ensures comparability with other GBD−based studies.

### 2.2 Methods

Data were extracted using the GBD Results Tool with the following parameters: location set to China; period from 1990 to 2023; measures selected as “Number” and “Rate”; and metrics including “Incidence,” “Prevalence,” “Deaths,” “Disability-adjusted life years (DALYs),” “Years Lived with Disability (YLDs),” and “Years of Life Lost (YLLs).” The population categories included “All Ages,” “Age-standardized,” and specific age groups ranging from “Under 5” to “95+ years.” Sex categories included “Female” and “Both.” Data retrieval occurred on January 6, 2026.

### 2.3 Study indicators and statistical analysis

This study characterized the epidemiology of cervical cancer using indicators such as incidence, prevalence, deaths, DALYs, YLDs, YLLs, and their corresponding rates. Age-standardized rates (ASRs) were applied to account for differences in population age structures across datasets, thereby enhancing comparability.

Joinpoint regression analysis was performed using Joinpoint Regression Software (Version 4.5.0, National Cancer Institute, USA). The optimal number of joinpoints was determined using the permutation test (with 4,499 permutations) at a significance level of 0.05. Annual percentage change (APC) and average annual percentage change (AAPC), along with their 95% confidence intervals (95% CI), were calculated to quantify trend direction and magnitude. A positive AAPC indicates an increasing trend from 1990 to 2023, whereas a negative AAPC indicates a decreasing trend. Changes were deemed statistically significant if the 95% CI excluded zero. Joinpoint regression model selection and assumptions: The maximum number of joinpoints was limited to five to avoid overfitting, given the 34-year study period. The model assumed that segment-specific log-linear trends were continuous at joinpoints and that errors were uncorrelated with constant variance. The Bayesian Information Criterion (BIC) was used to compare models with different numbers of joinpoints when the permutation test yielded ambiguous results.

An autoregressive integrated moving average (ARIMA) model was employed to forecast the age−standardized incidence rate (ASIR) and age−standardized death rate (ASDR) of cervical cancer in China for 2024–2038. Assumptions: The ARIMA model assumes that after appropriate differencing the series is stationary and that residuals are white noise. The modeling procedure consisted of four steps: Stationarity test: The Augmented Dickey−Fuller (ADF) test was applied to the original ASIR and ASDR series (1990–2023). If the series were non−stationary (p > 0.05), differencing was performed until stationarity was achieved. Model identification: After differencing, the autocorrelation function (ACF) and partial autocorrelation function (PACF) plots were examined to tentatively identify the orders of the autoregressive (p) and moving average (q) components. Model selection: Using the auto.arima function from the forecast package in R, candidate models were compared based on the corrected Akaike information criterion (AICc). For ASIR, the ARIMA(0,1,1) with drift model yielded the lowest AICc (1.61), outperforming alternatives such as ARIMA(1,1,0) (AICc = 3.24) and ARIMA(1,1,1) (AICc = 3.58). For ASDR, ARIMA(0,1,2) with drift was selected (AICc = –57.93), compared with ARIMA(0,1,1) (AICc = –55.01) and ARIMA(1,1,2) (AICc = –54.22). A drift term was allowed to capture the long-term downward trend. The Ljung–Box test was used to test whether the residuals were white noise (p > 0.05), and the ACF of residuals was inspected to ensure no significant autocorrelation remained. Only models passing these checks were used for forecasting. Because mortality rates cannot be negative, any lower bound of the 95% prediction interval falling below zero was truncated to zero for interpretation.

All statistical analyses and visualizations were performed using R software (Version 4.5.2).

## 3 Results

### 3.1 Trends in cervical cancer disease burden in China, 1990–2023

From 1990 to 2023, Chinese women exhibited divergent trends in cervical cancer disease burden indicators when comparing absolute numbers to age-standardized rates. Specifically, the absolute number of prevalent cases increased from approximately 446,000 to nearly 752,000; deaths rose from approximately 41,000 to 48,000; and new cases climbed from roughly 87,000 to 125,000. Correspondingly, the absolute values for DALYs and its components—YLLs and YLDs—also trended upward.

Conversely, age-standardized rates (per 100,000 population) declined as follows: ASDR decreased from 8.80 to 4.18; ASIR from 17.32 to 12.06; prevalence rate from 84.26 to 76.52; and DALYs rate from 294.14 to 132.72. Among the DALYs components, the age-standardized rate for YLLs decreased from 286.99 to 126.84, and for YLDs from 7.15 to 5.88 (**Table 1**).

**Table 1:** Changes in the burden of cervical cancer in China, 1990 to 2023.

Year	Number of prevalent cases	Age-standardized prevalence rate (per 100,000)	Number of deaths	Age-standardized mortality rate (per 100,000)	DALYs	DALYs Rate (per 100,000)
1990	445,795	84.26	41,076	8.8	1,457,115	294.14
2023	752,306	76.52	47,758	4.18	1,430,054	132.72
Change (%)	68.76%	-9.19%	16.27%	-52.50%	-1.86%	-54.87%
Year	YLDs	YLDs Rate (per 100,000)	YLLs	YLLs Rate (per 100,000)	Number of Incident Cases	Incidence Rate (per 100,000)
1990	36,740	7.15	1,420,375	286.99	86,808	17.32
2023	59,479	5.88	1,370,575	126.84	124,686	12.06
Change (%)	61.89%	-17.76%	-3.51%	-55.80%	43.64%	-30.37%

Among absolute indicators, prevalent cases showed the largest increase (+68.8%). In contrast, all key age-standardized rate indicators consistently decreased, with the mortality rate and DALYs rate exhibiting the most pronounced reductions of -52.5% and -54.9%, respectively.

### 3.2 Distribution of cervical cancer disease burden by age group in China, 2023

**Figure 1:**
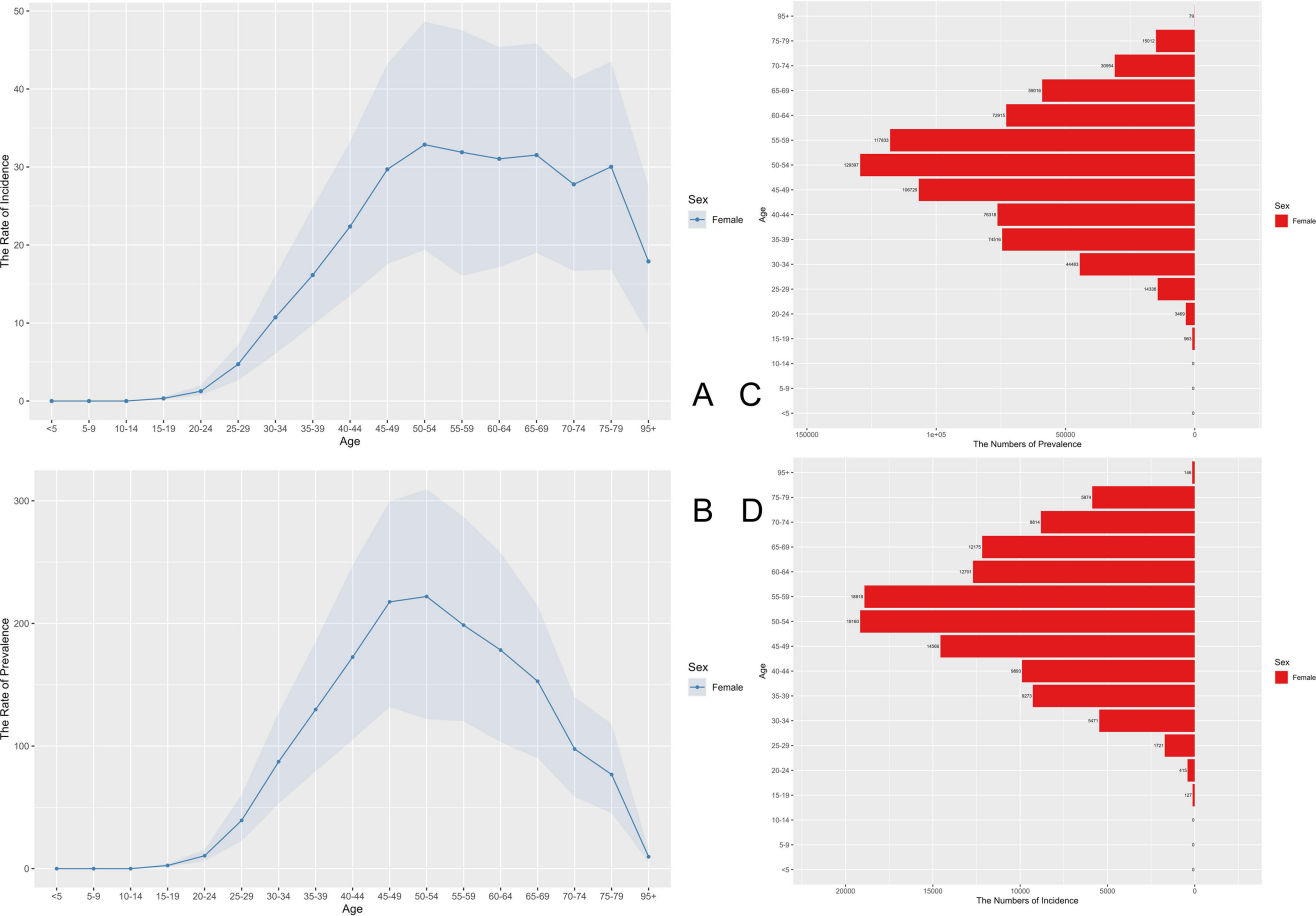
Stratified analysis of the disease burden of cervical cancer in different age groups in China in 2023. **(A)** Incidence rate; **(B)** Prevalence rate; **(C)** Number of incident cases; **(D)** Number of prevalent cases.

Data from 2023 indicate that the prevalence rate, number of prevalent cases, incidence rate, and new cases of cervical cancer in China increased significantly with age, with a markedly higher disease burden in women aged 55 years and older. The prevalence rate peaked in the 95+ age group, whereas the 5–9 age group exhibited the lowest rate. This distribution suggests the necessity of intensifying screening and early intervention efforts targeting middle-aged and elderly women (**Figure 1**). **Figure 1** illustrates the age−specific distribution of cervical cancer burden in 2023. The incidence and prevalence rates increased progressively with age, peaking in the oldest age groups. Notably, the number of incident cases and prevalent cases also rose sharply among women aged 55 years and older, highlighting the concentration of disease burden in this demographic.

### 3.3 Time Trend of cervical cancer disease burden in China, 1990–2023

**Figure 2:**
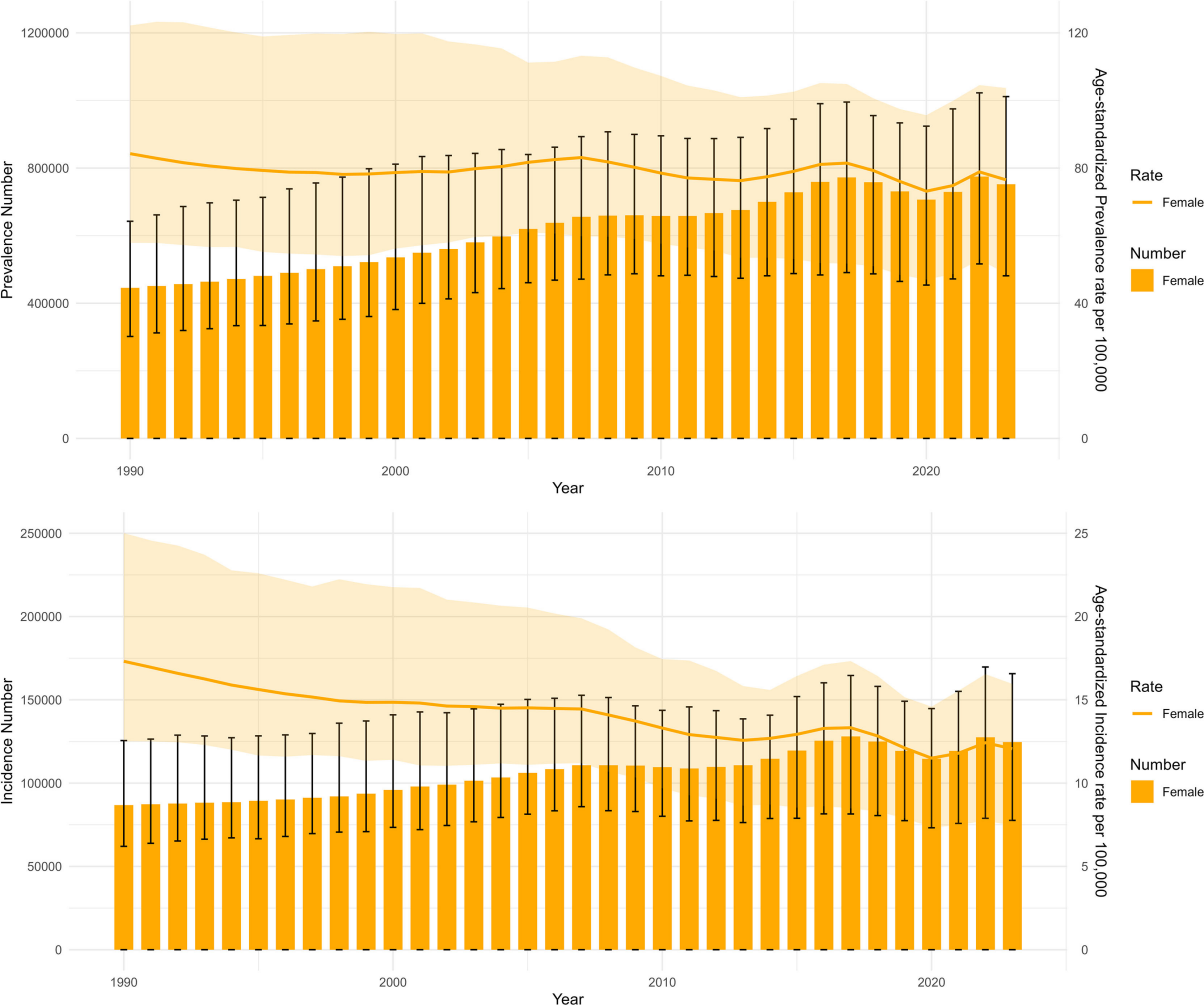
Time trend of disease burden of cervical cancer in China from 1990 to 2023.

Since 1990, absolute numbers of prevalent and incident cervical cancer cases displayed an overall fluctuating decline, with clear improvements post-2000. However, since approximately 2015, incidence rates have shown a slight resurgence in certain regions and specific age groups, particularly among older women. Although the overall disease burden has diminished, the heightened risk in older populations underscores the need for sustained and targeted cervical cancer prevention and control interventions, including enhanced screening and health education for the elderly demographic (**Figure 2**). **Figure 2** shows the temporal trends in absolute numbers and age−standardized rates of cervical cancer burden in China from 1990 to 2023. Absolute numbers increased steadily, while age−standardized rates declined, reflecting the dual burden of the disease. A slight resurgence in incidence was observed after 2015, particularly among older women, highlighting the need for continued vigilance.

### 3.4 Joinpoint regression model analysis of cervical cancer disease burden in China, 1990–2023

Joinpoint regression was applied to the age−standardized incidence rate (ASIR), age−standardized death rate (ASDR), and age−standardized DALYs rate of cervical cancer among Chinese women from 1990 to 2023. The results are summarized in **Table 2**.

**Table 2:** Joinpoint regression analysis of age−standardized incidence rate (ASIR), age−standardized death rate (ASDR), and age−standardized DALYs rate for cervical cancer among Chinese women, 1990–2023.

Indicator	Segment	Years	APC (95% CI)	AAPC (95% CI)
ASIR	1	1990–1997	–1.96 (–2.32 to –1.61)*	–1.02 (–1.31 to –0.73)*
	2	1997–2007	–0.45 (–0.68 to –0.22)*	
	3	2007–2013	–2.35 (–2.85 to –1.85)*	
	4	2013–2017	1.88 (0.74 to 3.03)*	
	5	2017–2020	–4.84 (–7.06 to –2.57)*	
	6	2020–2023	2.07 (0.73 to 3.43)*	
ASDR	1	1990–1996	–2.88* (—)	–2.14 (–2.57 to –1.71)*
	2	1996–2006	–1.87* (—)	
	3	2006–2013	–3.59* (—)	
	4	2013–2017	1.51* (—)	
	5	2017–2020	–6.64* (—)	
	6	2020–2023	1.34* (—)	
DALYs	1	1990–1996	–3.04* (—)	–2.38 (–2.84 to –1.91)*
	2	1996–2006	–1.94* (—)	
	3	2006–2013	–3.84* (—)	
	4	2013–2017	0.95* (—)	
	5	2017–2020	–6.20* (—)	
	6	2020–2023	1.15* (—)	

CI, confidence interval. *Indicates statistically significant trend (P < 0.05).

**Figure 3:**
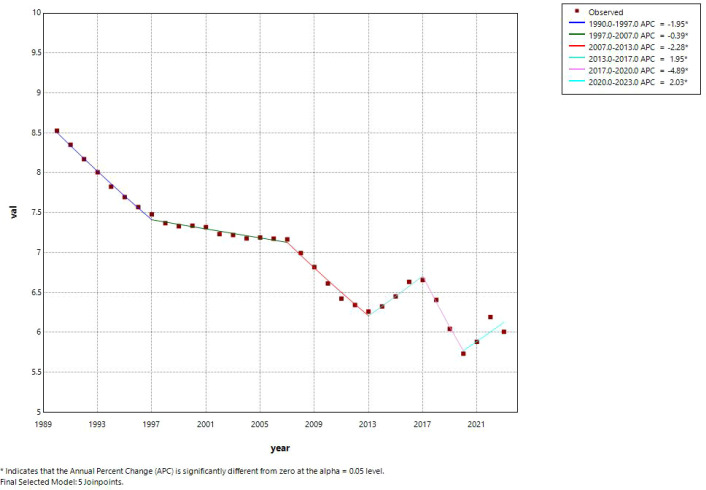
Joinpoint regression trend of the age−standardized incidence rate (ASIR) for cervical cancer among Chinese women, 1990–2023. The solid line represents the observed rates, the dashed line represents the fitted trend, and the red dots indicate the joinpoints identified by the permutation test (P < 0.05).

ASIR showed a significant overall downward trend with an average annual percent change (AAPC) of –1.02% (95% CI: –1.31% to –0.73%, P < 0.001). The trend comprised six distinct phases: a rapid decline from 1990 to 1997 (APC = –1.96%, 95% CI: –2.32% to –1.61%, P < 0.001), a slower decline from 1997 to 2007 (APC = –0.45%, 95% CI: –0.68% to –0.22%, P < 0.001), an accelerated decline from 2007 to 2013 (APC = –2.35%, 95% CI: –2.85% to –1.85%, P < 0.001), a brief rebound from 2013 to 2017 (APC = 1.88%, 95% CI: 0.74% to 3.03%, P = 0.002), a sharp decline from 2017 to 2020 (APC = –4.84%, 95% CI: –7.06% to –2.57%, P < 0.001), and a renewed increase from 2020 to 2023 (APC = 2.07%, 95% CI: 0.73% to 3.43%, P = 0.005). **Figure 3** illustrates this trend, showing the observed ASIR values, the fitted Joinpoint regression line, and the identified joinpoints (red dots). The six distinct phases are clearly visualized, with the recent rebound from 2020 to 2023 evident in the uptick at the end of the series.

**Figure 4:**
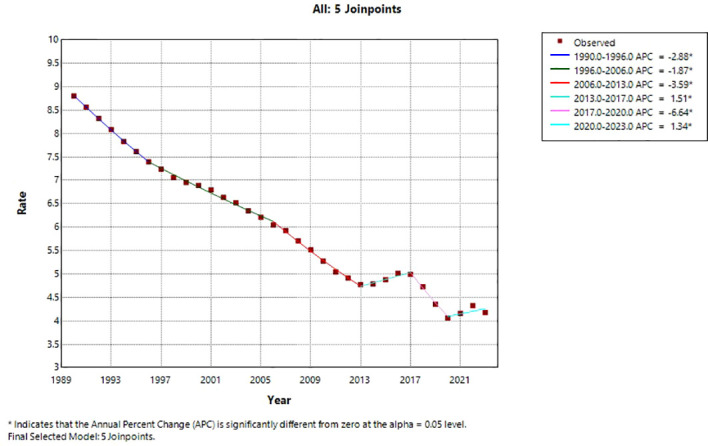
Joinpoint regression trend of the age−standardized death rate (ASDR) for cervical cancer among Chinese women, 1990–2023. The solid line represents the observed rates, the dashed line represents the fitted trend, and the red dots indicate the joinpoints identified by the permutation test (P < 0.05). Six joinpoints were identified, dividing the trend into six segments: 1990–1996, 1996–2006, 2006–2013, 2013–2017, 2017–2020, and 2020–2023.

ASDR also exhibited a significant overall decline (AAPC = –2.14%, 95% CI: –2.57% to –1.71%, P < 0.001). The trend consisted of six phases: a rapid decrease from 1990 to 1996 (APC = –2.88%, P < 0.001), a continued but slower decrease from 1996 to 2006 (APC = –1.87%, P < 0.001), an accelerated decrease from 2006 to 2013 (APC = –3.59%, P < 0.001), a brief increase from 2013 to 2017 (APC = 1.51%, P < 0.001), a sharp decline from 2017 to 2020 (APC = –6.64%, P < 0.001), and a slight rebound from 2020 to 2023 (APC = 1.34%, P < 0.001). **Figure 4** presents the Joinpoint regression trend for ASDR. Similar to ASIR, six phases were identified, with a pronounced sharp decline during 2017–2020 followed by a slight rebound from 2020 to 2023. The figure highlights the sustained long-term decline alongside recent fluctuations.

**Figure 5:**
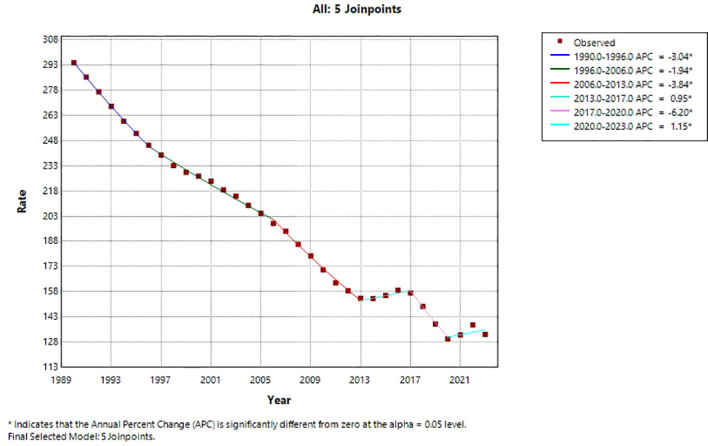
Joinpoint regression trend of the age-standardized DALYs rate for cervical cancer among Chinese women, 1990–2023. The solid line represents the observed rates, the dashed line represents the fitted trend, and the red dots indicate the joinpoints identified by the permutation test (P < 0.05). Six joinpoints were identified, dividing the trend into six segments: 1990–1996, 1996–2006, 2006–2013, 2013–2017, 2017–2020, and 2020–2023.

The age−standardized DALYs rate similarly declined significantly overall (AAPC = –2.38%, 95% CI: –2.84% to –1.91%, P < 0.001). Its six phases were: a rapid decline from 1990 to 1996 (APC = –3.04%, P < 0.001), a slower decline from 1996 to 2006 (APC = –1.94%, P < 0.001), an accelerated decline from 2006 to 2013 (APC = –3.84%, P < 0.001), a moderate increase from 2013 to 2017 (APC = 0.95%, P < 0.001), a steep decline from 2017 to 2020 (APC = –6.20%, P < 0.001), and a small increase from 2020 to 2023 (APC = 1.15%, P < 0.001). **Figure 5** displays the Joinpoint regression trend for the age−standardized DALYs rate. The pattern mirrors that of ASIR and ASDR, with a steep decline during 2017–2020 and a modest increase in the final segment, reflecting the overall consistency across these burden indicators.

### 3.5 Prediction of cervical cancer disease burden in China based on ARIMA model

**Figure 6:**
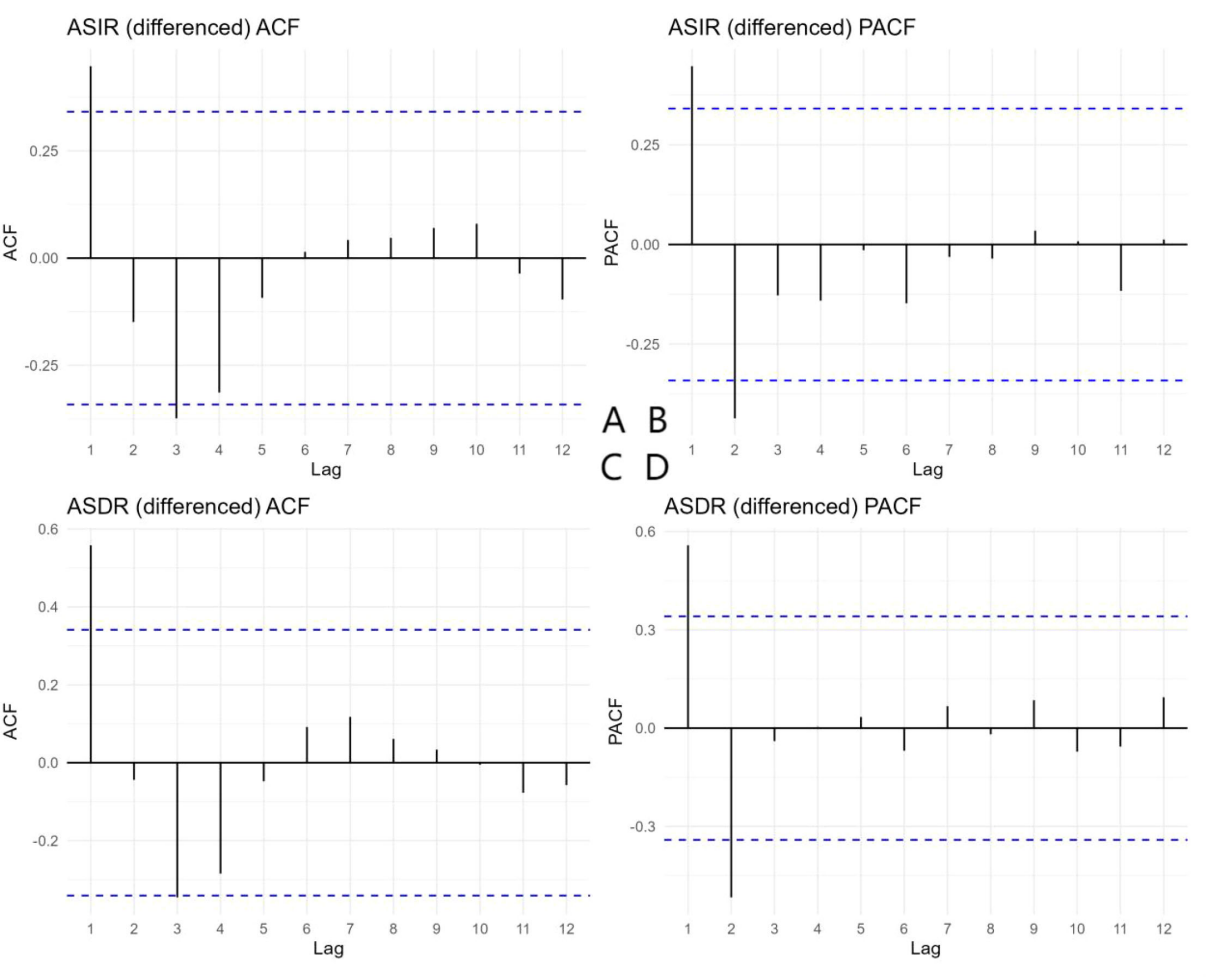
Autocorrelation function (ACF) and partial autocorrelation function (PACF) plots of the differenced age−standardized incidence rate (ASIR) and age−standardized death rate (ASDR) series for cervical cancer in China, 1990–2023. **(A)** ACF of differenced ASIR; **(B)** PACF of differenced ASIR; **(C)** ACF of differenced ASDR; **(D)** PACF of differenced ASDR. The blue dashed lines represent the 95% confidence intervals. The sharp cut−off of the ACF at lag 1 for ASIR suggests a moving average component of order 1, while the ACF of ASDR indicates possible orders 1 or 2.

**Figure 7:**
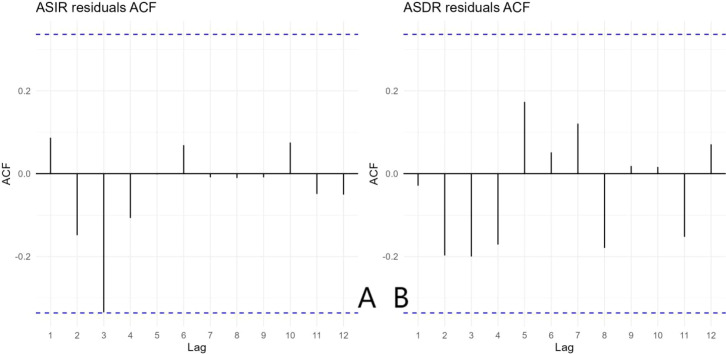
Residual ACF of the ARIMA(0,1,1) with drift model for ASIR; **(B)** Residual ACF of the ARIMA(0,1,2) with drift model for ASDR. All lags lie within the 95% confidence intervals (blue dashed lines), indicating white noise and confirming that both models adequately capture the underlying structure of the data.

The ADF test indicated that the original ASIR series was non−stationary (p = 0.176). After first−order differencing, the series became stationary (p < 0.05). The ACF and PACF plots of the differenced ASIR series (**Figures 6A,B**) showed a sharp cut−off at lag 1, supporting the selection of an MA(1) component. Based on AICc minimization, the optimal model was ARIMA(0,1,1) with drift (AICc = 1.61). Residual diagnostics (**Figure 7**) confirmed that no significant autocorrelation remained, indicating adequate model specification. The Ljung--Box test on the residuals gave Q = 6.22 (df = 6, p = 0.399), confirming white noise.

For ASDR, the original series was also non−stationary (p = 0.528) and became stationary after first differencing (p < 0.05). The ACF and PACF of the differenced ASDR (**Figures 6C, D**) indicated possible q = 1 or 2. The best model was ARIMA(0,1,2) with drift (AICc = –57.93). Residual diagnostics yielded Q = 6.33 (df = 5, p = 0.275), confirming model adequacy. Both models therefore provided a reliable basis for forecasting. Predictions derived from the ARIMA time series model are presented in **Table 3**. During 2024–2038, the ASIR of cervical cancer in China is projected to decline gradually, whereas the ASDR is expected to decline sharply.

**Table 3:** ARIMA Model Prediction of Age-Standardized Incidence Rate (ASIR) and Age-Standardized Death Rate (ASDR) of Cervical Cancer in China, 2024 to 2038.

Year	Age-standardized incidence rate (ASIR, per 100,000) mean (95% *CI*)	Age-standardized mortality rate (ASDR, per 100,000) mean (95% *CI*)
2024	11.14 (10.74–11.54)	3.70 (3.54–3.87)
2025	10.48 (9.65–11.31)	3.18 (2.77–3.59)
2026	10.45 (9.35–11.54)	2.91 (2.21–3.61)
2027	10.75 (9.56–11.93)	2.90 (1.96–3.85)
2028	10.86 (9.64–12.07)	2.94 (1.80–4.09)
2029	10.58 (9.33–11.82)	2.78 (1.43–4.12)
2030	10.10 (8.78–11.42)	2.39 (0.81–3.96)
2031	9.75 (8.32–11.19)	1.96 (0.09–3.84)
2032	9.66 (8.12–11.19)	1.71 (0.00–3.93)
2033	9.67 (8.07–11.27)	1.64 (0.00–4.19)
2034	9.60 (7.96–11.23)	1.59 (0.00–4.45)
2035	9.37 (7.68–11.05)	1.41 (0.00–4.58)
2036	9.07 (7.33–10.81)	1.08 (0.00–4.59)
2037	8.84 (7.03–10.64)	0.72 (0.00–4.61)
2038	8.70 (6.83–10.57)	0.48 (0.00–4.76)

For ASDR predictions from 2032 to 2038, the original 95% CI lower bounds were negative (e.g., -0.51 to -3.80). These were truncated to 0.00 because mortality rates cannot be negative. The upper bounds remain unchanged.

The predicted mean ASIR (per 100,000) is anticipated to decrease from 11.14 in 2024 to 8.70 in 2038, representing a cumulative reduction of approximately 21.9%. Concurrently, the predicted mean ASDR is forecasted to drop markedly from 3.70 to 0.48, a cumulative decrease of 87.0%.

**Figure 8:**
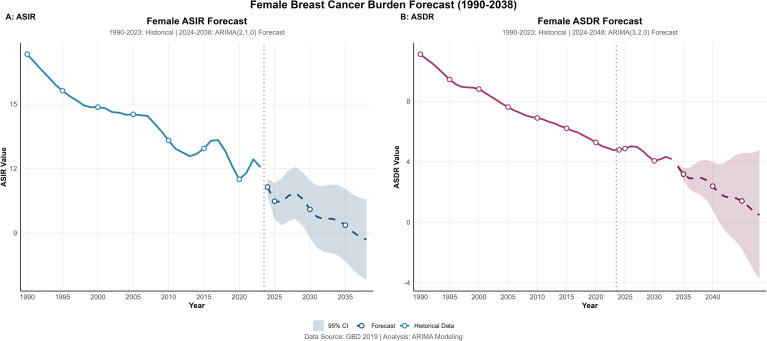
**(A)** Fitting prediction of ASIR of cervical cancer in China based on ARIMA model; **(B)** Fitting prediction of ASDR of cervical cancer in China based on ARIMA model, 1990–2038.

Based on this trend model, it is projected that from 2024 to 2038, both ASIR and ASDR of cervical cancer among Chinese women will continue to decline; ASIR is expected to decrease from 11.14 (95% CI, 10.74–11.54) in 2024 to 8.70 (95% CI, 6.83–10.57) in 2038. Meanwhile, ASDR is predicted to fall substantially from 3.70 (95% CI, 3.54–3.87) in 2024 to 0.48 (95% CI, 0.00–4.76) in 2038, with the lower bound truncated at zero as negative mortality rates are not biologically plausible. The wide confidence interval reflects increasing statistical uncertainty over longer forecast horizons (**Table 3**) (**Figure 8**).

## 4 Discussion

We analyzed long-term trends in cervical cancer burden in China (1990–2023). The key finding is a divergence: absolute numbers of prevalent cases, deaths, and new cases increased, whereas all age-standardized rates (incidence, mortality, prevalence, DALYs) declined. This duality reflects both progress and emerging challenges in China’s cervical cancer control.

The sustained decline in age-standardized rates signals a public health success, driven by expanded screening, better diagnosis and treatment, and recently, HPV vaccination. The sharp drops in mortality (–52.5%) and DALYs (–54.9%) are especially notable, showing substantial progress in reducing premature death.

Conversely, rising absolute numbers—prevalent cases up 68.8%, new cases up 43.6%—highlight growing service demands from population aging and growth. China’s large population(6) remains at risk, requiring sustained investment in screening, diagnosis, and treatment. Thus, while continuing to reduce mortality, China must also manage the rising absolute burden of patients needing long−term follow−up and survivorship care.

The age-specific distribution observed in 2023—with disease burden concentrated in women aged 55 years and older—has important implications for cervical cancer prevention in China. This pattern reflects a combination of historical exposure, cohort effects, and current intervention coverage gaps. Women currently aged ≥55 years largely missed universal HPV vaccination, as the first HPV vaccine was not introduced in China until 2016, and nationwide vaccination programs remain limited. Consequently, this population has acquired HPV infections primarily through natural exposure, with a non−negligible proportion progressing to cervical lesions or invasive cancer over time. Moreover, cervical cancer screening coverage, although improved, remains suboptimal among middle−aged and older women, especially in rural and resource−limited areas. National data show that participation rates decline with age, and many older women have never been screened or have not undergone regular screening, leading to missed opportunities for early detection. From a prevention perspective, this age−concentrated burden underscores two priorities. First, it highlights the need to strengthen secondary prevention by actively reaching older unscreened women through community−based outreach, optimized screening protocols, and integration with routine primary care. Second, it reinforces the importance of expanding primary prevention among younger cohorts to prevent a similar burden as they age. Additionally, age−related physiological and immunological changes—such as postmenopausal alterations in the vaginal microenvironment and immune senescence—may further influence HPV persistence and disease progression, warranting tailored management strategies. Thus, interpreting the age distribution as a reflection of intervention gaps and biological vulnerability provides a concrete basis for refining China’s cervical cancer elimination strategy (6), aligning with the “90–70–90” targets set by the national action plan (7).

Joinpoint regression revealed incidence fluctuations, with notable rebounds in 2013–2017 and 2020–2023. The 2013–2017 rebound occurred around the time of HPV vaccine introduction in China. Although the bivalent vaccine was approved in 2016, followed by quadrivalent (2017) and nonavalent (2018) vaccines, supply was severely constrained by reliance on imports and strict batch release quotas. Vaccination costs were high and not covered by the National Immunization Program, resulting in negligible population coverage (8). The observed ASIR rise during these years may be partly explained by expanded use of sensitive HPV DNA testing in urban screening programs, which could have increased case detection, rather than a true rise in infection risk alone. Changes in sexual behavior among younger cohorts cannot be ruled out. The 2020–2023 rebound temporally overlapped with the COVID−19 pandemic. Widespread lockdowns and healthcare disruptions likely caused delays in routine screening and diagnostic follow−up, creating a backlog of detected cases that temporarily elevated reported incidence when services resumed. Pandemic−related interruptions also hindered HPV vaccination rollout. Although a domestic bivalent vaccine became available in 2020, its cumulative impact on incidence reduction remained limited during this period. This rebound highlights the vulnerability of cancer control programs to public health emergencies and the need for resilient health systems (9).

Our findings align with global GBD 2023 trends, which showed that age−standardized cervical cancer mortality declined by approximately 50% worldwide between 1990 and 2023, with the largest reductions in countries with organized screening and HPV vaccination programs (5). However, the pattern of rising absolute numbers alongside declining rates is more pronounced in China due to its large and rapidly aging population. Compared with other middle−income countries such as Brazil and India, China has achieved comparable or greater reductions in age−standardized mortality, but absolute burden growth presents unique challenges requiring context−specific strategies (10, 11). Within China, our findings are consistent with regional studies from urban centers such as Beijing and Shanghai, which have reported declining screening−adjusted incidence rates since the early 2000s (12). The present national−level analysis extends previous work by quantifying the dual trend over a longer period (1990–2023), identifying incidence fluctuations via Joinpoint regression, and providing ARIMA projections to 2038. The concentration of burden in women aged ≥55 years, suggested in provincial studies, is now confirmed nationally with detailed epidemiological interpretation linked to vaccination gaps and screening patterns. Collectively, these contributions provide a comprehensive, up−to−date baseline for optimizing China’s cervical cancer elimination strategy.

ARIMA model predictions indicate that from 2024 to 2038, both ASIR and ASDR are expected to continue declining, with mortality showing particularly substantial reductions. This trend aligns with expected expansions in HPV vaccination coverage, broader screening, and advances in diagnosis and treatment. However, the relatively gradual decline in incidence underscores the need for sustained, long−term efforts to reduce new cases and for sensitive surveillance to detect trend changes.

Based on our core findings—(1) the dual trend of rising absolute burden with declining age−standardized rates, (2) burden concentration in women ≥55 years, and (3) fluctuating incidence with recent rebounds—we propose the following differentiated strategies:

1. Targeted screening for women aged ≥55 years. Given their high burden and historically low screening participation, we recommend age−adapted protocols. For women aged 55–65 years without regular screening, catch−up programs should be prioritized through community outreach and integration with primary care. For those aged ≥65 years with adequate prior screening, risk−based cessation criteria should be refined. Primary HPV testing with genotyping should be expanded in this population for better risk stratification.
2. Remedial measures for prevention gaps during rebound periods. The rebounds in 2013–2017 and 2020–2023 reveal system vulnerabilities. We recommend: (a) real−time surveillance to detect incidence fluctuations promptly; (b) ensuring resilience of prevention services during public health emergencies (e.g., maintaining screening and vaccination during pandemics); and (c) accelerating HPV vaccination integration into the National Immunization Program to achieve high, equitable coverage among adolescent girls.
3. Long−term strategies in the context of population aging. Rising absolute burden due to aging requires a shift from focusing solely on mortality reduction to comprehensive care. This includes: (a) expanding screening infrastructure and workforce; (b) strengthening health systems for diagnosis, treatment, and long−term follow−up, especially for older women with comorbidities; and (c) integrating cervical cancer prevention into elderly health management programs using existing platforms (e.g., basic public health services) to reach less−screened older women.
4. Synergizing primary and secondary prevention across age cohorts. For younger cohorts, sustained efforts to achieve the “90–70–90” targets (90% HPV vaccination among adolescent girls, 70% screening coverage, 90% treatment) are essential to prevent future burden. For middle−aged and older cohorts, strengthening secondary prevention through accessible, high−quality screening and prompt treatment remains the priority. Aligning strategies with age−specific needs will help China accelerate progress toward cervical cancer elimination while managing the growing absolute burden.

Furthermore, while our findings are specific to China, they offer lessons for other low- and middle-income countries (LMICs) facing similar epidemiological transitions. The dual pattern of declining age-standardized rates but rising absolute burden is likely to emerge in LMICs with expanding screening coverage, limited HPV vaccination, and aging populations. The concentration of disease burden in women aged ≥55 years—who largely missed vaccination opportunities—is also relevant to many LMICs where older cohorts remain unscreened and unvaccinated. Moreover, the observed incidence rebounds following health system disruptions (e.g., during COVID-19) highlight the vulnerability of cancer prevention programs in resource-constrained settings, underscoring the need for resilient service delivery models. A 2022 systematic review of cervical cancer screening in LMICs confirmed widespread barriers across individual, cultural, health system, and structural levels, and called for urgent policy implementation, enhanced health system capacity, community-based education and information dissemination, as well as stronger policies supporting women’s health and gender equity, to effectively address the inequitable burden of cervical cancer in LMICs (13). However, specific policy recommendations (e.g., HPV vaccination integration, age-adapted screening) should be adapted to local health system capacity, cultural contexts, and economic constraints. Thus, while China’s experience provides valuable insights, each LMIC must tailor its cervical cancer elimination strategy to its own realities.

Nevertheless, several limitations should be noted. First, regarding the GBD data, China-specific estimates rely on multiple sources (e.g., vital registration, disease surveillance points, hospital records) that vary in completeness and data quality across regions and time periods. Underreporting, particularly in rural or less developed areas, may lead to underestimation of cervical cancer burden in certain subgroups. Additionally, GBD employs statistical modeling to fill data gaps, including covariate adjustment and spatial–temporal smoothing, which introduces uncertainty not fully captured by the reported confidence intervals. The accuracy of cause-of-death attribution for cervical cancer also depends on local death certification and coding practices, which may differ across provinces. Second, regarding our study’s own limitations, our analysis focuses exclusively on China, so the findings may not be directly generalizable to other countries without contextual adaptation. The ARIMA predictions have wider confidence intervals over longer forecast horizons, and the truncation of negative mortality bounds, while biologically necessary, reflects inherent statistical uncertainty. Furthermore, due to data availability, we did not stratify by HPV genotype, urban versus rural residence, or socioeconomic status, which could mask important within-country disparities. Despite these limitations, this study provides a robust national-level assessment of cervical cancer burden trends in China and offers evidence to inform prevention strategies.

## Data Availability

The datasets presented in this study can be found in online repositories. The names of the repository/repositories and accession number(s) can be found in the article/supplementary material.
